# Impact of gallstone disease on the risk of stroke and coronary artery disease: evidence from prospective observational studies and genetic analyses

**DOI:** 10.1186/s12916-023-03072-6

**Published:** 2023-09-13

**Authors:** Li Zhang, Wenqiang Zhang, Lin He, Huijie Cui, Yutong Wang, Xueyao Wu, Xunying Zhao, Peijing Yan, Chao Yang, Changfeng Xiao, Mingshuang Tang, Lin Chen, Chenghan Xiao, Yanqiu Zou, Yunjie Liu, Yanfang Yang, Ling Zhang, Yuqin Yao, Jiayuan Li, Zhenmi Liu, Chunxia Yang, Xia Jiang, Ben Zhang

**Affiliations:** 1 Department of Epidemiology and Biostatistics, Institute of Systems Epidemiology, and West China-PUMC C. C. Chen Institute of Health, West China School of Public Health and West China Fourth Hospital, Sichuan University, No. 16, Section 3, South Renmin Road, Wuhou District, Chengdu, 610041 China; 2 Department of Maternal, Child and Adolescent Health, West China School of Public Health and West China Fourth Hospital, Sichuan University, Chengdu, China; 3 Department of Iatrical Polymer Material and Artificial Apparatus, School of Polymer Science and Engineering, Sichuan University, Chengdu, China; 4 Department of Occupational and Environmental Health, West China School of Public Health and West China Fourth Hospital, Sichuan University, Chengdu, China; 5 Department of Nutrition and Food Hygiene, West China School of Public Health and West China Fourth Hospital, Sichuan University, Chengdu, China; 6 Department of Clinical Neuroscience, Karolinska Institutet, Stockholm, Sweden

**Keywords:** Gallstone disease, Cardiovascular disease, Phenotypic association, Genetic correlation, Mendelian randomization

## Abstract

**Background:**

Despite epidemiological evidence associating gallstone disease (GSD) with cardiovascular disease (CVD), a dilemma remains on the role of cholecystectomy in modifying the risk of CVD. We aimed to characterize the phenotypic and genetic relationships between GSD and two CVD events – stroke and coronary artery disease (CAD).

**Methods:**

We first performed a meta-analysis of cohort studies to quantify an overall phenotypic association between GSD and CVD. We then investigated the genetic relationship leveraging the largest genome-wide genetic summary statistics. We finally examined the phenotypic association using the comprehensive data from UK Biobank (UKB).

**Results:**

An overall significant effect of GSD on CVD was found in meta-analysis (relative risk [RR] = 1.26, 95% confidence interval [CI] = 1.19–1.34). Genetically, a positive shared genetic basis was observed for GSD with stroke (${r}_{g}$=0.16, *P* = 6.00 × 10^–4^) and CAD (${r}_{g}$=0.27, *P* = 2.27 × 10^–15^), corroborated by local signals. The shared genetic architecture was largely explained by the multiple pleiotropic loci identified in cross-phenotype association study and the shared gene-tissue pairs detected by transcriptome-wide association study, but not a causal relationship (GSD to CVD) examined through Mendelian randomization (MR) (GSD-stroke: odds ratio [OR] = 1.00, 95%CI = 0.97–1.03; GSD-CAD: OR = 1.01, 95%CI = 0.98–1.04). After a careful adjustment of confounders or considering lag time using UKB data, no significant phenotypic effect of GSD on CVD was detected (GSD-stroke: hazard ratio [HR] = 0.95, 95%CI = 0.83–1.09; GSD-CAD: HR = 0.98, 95%CI = 0.91–1.06), further supporting MR findings.

**Conclusions:**

Our work demonstrates a phenotypic and genetic relationship between GSD and CVD, highlighting a shared biological mechanism rather than a direct causal effect. These findings may provide insight into clinical and public health applications.

**Supplementary Information:**

The online version contains supplementary material available at 10.1186/s12916-023-03072-6.

## Background

Gallstone disease (GSD) and cardiovascular disease (CVD) are both common and costly global public health issues, of which comorbidity has long been documented [1–4]. In a large meta-analysis of prospective studies involving over 1.2 million participants, an increased risk of CVD events has been reported among GSD patients (pooled hazard ratio [HR] = 1.23, 95% confidence interval [CI] = 1.16–1.30) [5]. Despite epidemiological evidence associating GSD with CVD, a critical clinical dilemma remains on the uncertainty being present over the role of cholecystectomy – the gold standard therapy for symptomatic GSD – in modifying the risk of CVD. By performing cholecystectomy, the deleterious effect of GSD on CVD risk has not been counteracted (compared to GSD patients without undergoing cholecystectomy) but rather increased (compared to general populations) [6]. Despite being drawn from observational studies rather than randomized controlled trials, this piece of evidence still reflects a potentially complex etiological interplay. It’s essential to acknowledge that these findings may be biased by distinct patient and disease characteristics between patients who undergo cholecystectomy versus those do not. Indeed, phenotypic associations derived from conventional observational designs can be subject to bias, confounding, and reverse causality, while limited accessibility to study samples or covariates further hinders an accurate quantification of effects [4, 7, 8]. One way of addressing the discrepancies between epidemiological and clinical results is to investigate the genetic underpinnings of related traits [9].

Both GSD and CVD are known to under genetic influences, with SNP (single-nucleotide polymorphisms) -heritability estimates of 25% and 40% [10–13], as well as a considerable number of disease-associated variants (GSD: *N* = 62; stroke: *N* = 23; coronary artery disease (CAD): *N* = 241) elucidated by recent genome-wide association studies (GWAS) [14–16]. Multiple common genetic loci (i.e., *CYP7A1, NPC1L1, ABCG5/8, APOE,* and *FABP2*) affecting both traits have also been identified, suggesting a potential intrinsic link underlying GSD and CVD [17–20].

**Fig. 1:**
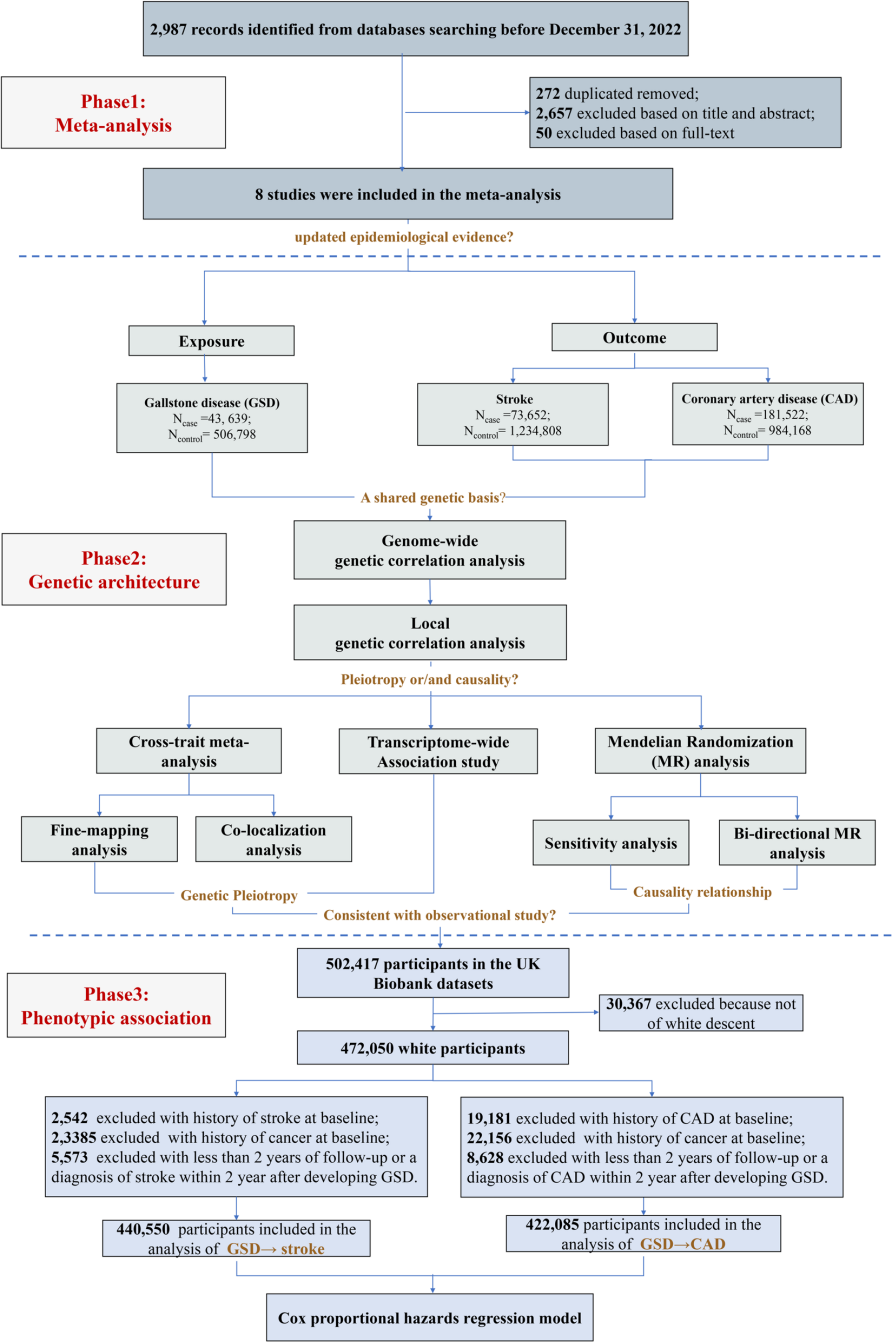
Flowchart on the overall study design. Gallstone disease was set as exposure, and two CVD phenotypes (stroke and coronary artery disease) were included as outcomes. We first updated the epidemiological evidence between GSD and CVD. We then performed a comprehensive genome-wide cross-trait analysis to investigate the shared genetic architecture underlying both traits and dissected such shared genetic basis into pleiotropy and causality. Lastly, we used UK Biobank (UKB) data to explore the phenotypic association

To better elucidate the relationship between GSD and CVD, we first meta-analyzed currently available epidemiological investigations of prospective design on GSD (or cholecystectomy) and incident CVD to quantify a crude overall phenotypic association. We then leveraged large-scale genome-wide genetic data as well as a complied analytical strategy – genome-wide cross-trait analysis – to determine shared and distinct genetic architecture. Such analysis features in several analytic aspects: genetic correlation analysis to estimate overall and local genetic correlation, cross-trait meta-analysis and transcriptome-wide association study (TWAS) to identify potential pleiotropic loci, and Mendelian randomizations (MR) to make causal inferences [9]. Finally, capitalizing information from a large cohort of 0.5 million individuals (the UK Biobank study) with full accessibility to important confounders, we carefully examined the phenotypic association between GSD and CVD. Leveraging these comprehensive genetic and observational data, our study aimed to extensively dissect the genetic and phenotypic relationships between GSD and CVD to inform clinical and public health interventions. The overall study design is shown in Fig. 1.

## Methods

### GWAS data sets

#### GSD GWAS

The hitherto largest GWAS of GSD was obtained from meta-analyzing data of UK Biobank (UKB) and FinnGen, comprising 550,437 European individuals (43,639 cases and 506,798 controls) [14]. Independent top-associated SNPs reaching genome-wide significance (*P* < 5 × 10^–8^) after removing SNPs in LD (r^2^ > 0.25 across a 250 kb window) were identified. We extracted relevant information of 62 GSD-associated index SNPs for instrumental variables (IVs) as well as downloaded full set summary statistics. The effect size and relevant information of GSD IVs are shown in Additional file 1: Table S1.

#### CVD GWAS

Two most common CVD events, stroke and CAD, were included in our study. GWAS summary data of stroke was obtained from the multi-ancestry meta-analyzing data of GIGASTROKE Consortium, among which 1,308,460 were European individuals (73,652 cases and 1,234,808 controls) [15]. This meta-GWAS identified 23 independent genome-wide significant SNPs at a *P*-threshold of 5 × 10^–8^. GWAS summary data of CAD was conducted meta-analyzing data across nine studies, totaling 181,522 cases and 984,168 controls of predominantly European ancestry (> 95%) [16]. This meta-GWAS identified 241 significant independent SNPs (*P* < 5 × 10^–8^). For both GWASs, we extracted relevant information of these trait-associated index SNPs (23 stroke-associated index SNPs and 241 CAD-associated index SNPs) for reverse Mendelian randomization (MR) analysis as well as downloaded full set summary statistics for genome-wide cross traits analysis.

A table detailing relevant information of each included dataset is presented (Additional file 1: Table S2). To conduct subsequent analysis, we performed pre-processing and quality control procedures on summary statistics as follows: 1) Retrieval of SNP (rs) ids; 2) Computation of Z-scores using log (OR)/SE when these were not available; 3) Exclusion of indels; and 4) Removal of duplicate SNPs. After applying variant filtering, the number of variants left for analysis was 10,064,832 for GSD; 7,650,286 for stroke; and 20,680,288 for CAD.

### UK Biobank data

UKB is a large community-based cohort study involving more than 500,000 individuals aged 40 and 69 years across the UK between 2006 and 2010 [21]. At recruitment, all participants gave informed consent to participate and be followed up, among which we only considered 472,050 participants of white descent. We set GSD as exposure, ascertained as any hospital admission with an International Classification of Diseases, Ninth Revision (ICD- 9) codes or Tenth Revision (ICD-10) codes relating to GSD (ICD-9 codes 574–576, 997, and 560; ICD-10 codes K80, K81, K85, K91, and K56) or with cholecystectomy Operative Procedures (OPCS) codes (OPCS 3 codes 522; OPCS4 codes J18) [14]. We focused on two most common CVD events – stroke and CAD as outcomes, which were defined as ICD-9 codes (430, 431, 433, 434, and 436) and ICD-10 codes (I60, I61, I63, and I64) for stroke, as well as ICD-9 codes (410–414) and ICD-10 codes (I20-I25) for CAD.

We excluded participants with a history of CVD or cancer at baseline. Participants with less than two years of follow-up or a diagnosis of CVD within 2 years after developing GSD were also excluded to ensure a research-quality follow-up and to reduce the possibility of reverse causation. In total, 440,550 participants were included in the GSD-stroke analysis and 422,085 in the GSD-CAD analysis.

Regarding the reverse association, we excluded participants with history of GSD or cancer at baseline. Participants with less than two years of follow-up or a diagnosis of GSD within 2 years after developing CVD were also excluded, finally leaving 431,904 participants in the stroke-GSD analysis and 429,105 in the CAD-GSD analysis.

### Statistical analysis

#### Meta-analysis

To obtain the most current epidemiological evidence, we conducted a meta-analysis incorporating results of previously published cohort studies. Referring to the Preferred Reporting Items for Systematic Reviews and Meta-Analysis Protocols [22] and the Meta-analysis of Observational Studies in Epidemiology [23], we first conducted a systematic literature search before December 31, 2022. The detailed search strategy was shown in Additional file 1: Table S3. Articles were considered for inclusion if: (1) published in a peer-reviewed journal in English; (2) nested case–control or cohort study design; (3) the exposure was GSD defined as the presence of gallstones or history of cholecystectomy, and the outcome of interest was CVD incidence (rather than death) including stroke, CAD, and other cardiovascular events; (4) risk estimates and their corresponding 95%CIs were reported. We excluded studies that were not published as full research articles (such as conference abstracts) or provided insufficient data.

We extracted the following information from each eligible publication: author, year, region, study design, sample size, Female (%), numbers of GSD, GSD diagnosis, CVD definition, years of follow-up, confounders adjustment, effect size, and quality assessment. The Newcastle–Ottawa Scale was used to evaluate the quality of included studies [24], ranging from score 1 (lowest quality) to score 9 (highest quality). The Cochran *Q* test and *I*^*2*^ statistic were used to assess heterogeneity among the included studies [25]. We pooled estimates using random-effects inverse-variance model after accounting for heterogeneity between studies [26]. To explore the source of heterogeneity, we conducted subgroup analysis on the basis of follow-up years, sex, study design, and GSD diagnosis. Sensitivity analysis were performed to estimate the robustness of results by omitting one study at a time [27]. All analyses were undertaken in R version 3.3.6 using the “meta” package.

#### Global and local genetic correlation analyses

We performed pairwise genetic correlation analysis using cross-trait linkage disequilibrium score regression (LDSC) [28] and genetic covariance analyzer (GNOVA) [29]. For LDSC, we used pre-computed linkage disequilibrium (LD) scores derived from ~ 1.2 million common SNPs in European ancestry represented in the HapMap3 reference panel excluding the HLA region. We also utilized a complementary method GNOVA to estimate genetic correlations, which demonstrated comparable robustness to LDSC [30]. Both analyses could control for potential sample overlap, ensuring reliable results. We applied quality-control steps by using the script munge_sumstats.py in both analyses. The genetic correlation estimate (${r}_{g}$) ranges from –1 to + 1, with –1 indicating a perfect negative correlation, and + 1 indicating a perfect positive correlation. For both analyses, a Bonferroni-corrected *P*-value (*P* < 0.05/2, number of CVD phenotypes) was used to define statistical significance.

We estimated the pairwise local genetic correlation using SUPERGNOVA [31]. This algorithm partitions the whole genome into approximately 2,353 LD-independent blocks and provides a precise quantification of the similarity between pairs of traits driven by genetic variations at each region. A Bonferroni-corrected *P*-value (*P* < 0.05/2,353) was used to define statistical significance.

#### Cross-trait meta-analysis

We next conducted a Cross-Phenotype Association (CPASSOC) analysis to identify potential pleiotropic loci [32]. CPASSOC provides two estimates, namely S_Hom_ and S_Het_, which effectively combine summary statistics across traits controlling for population structure and cryptic relatedness. While S_Hom_ is the most powerful when genetic effect sizes are homogeneous, this assumption is often violated in the context of meta-analyzing multiple traits. As an extension of S_Hom_, S_Het_ maintains statistical power even in the presence of heterogeneity by assigning greater weights to larger trait-specific effect sizes. Therefore, we adopted the S_Het_ estimate in our analysis. We applied PLINK clumping function to obtain independent SNPs (parameters: –clump-p1 5e-8 –clump-p2 1e-5 –clump-r2 0.2 –clump-kb 500) [33]. Among each trait pair, significant shared SNPs were defined as index variants satisfying *P*_CPASSOC_ < 5 × 10^–8^ and *P*_single-trait_ < 1 × 10^–3^ (for both traits). Particularly, a significant pleiotropic SNP satisfies the following conditions was further considered as a novel shared SNP: (1) was not driven by any single trait (5 × 10^–8^ < *P*_single-trait_ < 1 × 10^–3^); and (2) was not in LD (r^2^ < 0.1) with any previously reported genome-wide significant SNPs of single-trait, and none of their neighboring SNPs (± 500 kb) reached *P* < 5 × 10^–8^ in single-trait GWASs.

We used Ensemble Variant Effect Predictor (VEP) [34] for detailed functional annotation of identified significant pleiotropic SNPs. This approach facilitated the identification of candidate genes based on physical proximity to the pleiotropic SNPs, providing valuable insights into their functional implications.

#### Fine-mapping credible set and colocalization analysis

We conducted a fine-mapping analysis using FM-summary (https://github.com/hailianghuang/FMsummary) to identify a credible set of SNPs that were 99% likely to contain the causal SNP at each of the significant shared SNPs obtained from CPASSOC. This algorithm maps the primary signal and uses a flat prior with the steepest descent approximation, assuming at least one causal variant exists within a given region [35].

We performed a colocalization analysis using Coloc [36]. We extracted summary statistics for variants within 500 kb of the index SNP at each shared locus, calculated the posterior probability for H4 (PPH4, the probability that both traits associated through sharing a single causal variant) and H3 (PPH3, the probability that the two traits associated with different causal variants). A locus was considered colocalized if PPH4 was greater than 0.8.

#### Transcriptome-wide association study

To identify associations with regard to transcriptome gene expression in specific tissues, we conducted a transcriptome-wide association study (TWAS) using FUSION (http://gusevlab.org/projects/fusion/) based on 49 Genotype-Tissue Expression (GTEx) version 8 tissue expression weights [37]. We first performed 49 TWASs for each trait and then intersected single-trait TWAS results to determine the shared gene-tissue pairs across traits. Bonferroni correction was applied within each tissue to account for multiple comparisons.

#### Mendelian randomization analysis

We next conducted two-sample MR analysis to detect the putative causal relationship between GSD and CVD, and reported study design according to STROBE-MR (Additional file 1: Table S4) [38]. Causal Analysis Using Summary Effect Estimates (CAUSE) was applied as our primary approach [39]. Complementary to CAUSE, inverse-variance weighted (IVW), MR-Egger regression and weighted median approach were performed to examine the robustness and consistency of results. We applied a Bonferroni correction to account for multiple comparisons in our MR analysis. A *P*-threshold of 0.05/2 (number of CVD phenotypes) was defined as statistical significance.

We conducted several important sensitivity analyses to validify MR results, including (i) the exclusion of palindromic IVs [40]; (ii) the exclusion of pleiotropic IVs; and (iii) the reverse-direction MR to rule out reverse causality. An effect estimate was determined as putative causal if it was statistically significant in CAUSE and remained directionally consistent across other analyses. MR analyses were conducted using packages CAUSE (version 1.2.0) and TwoSampleMR (version 0.5.6) in R (version 4.2.1).

#### Observational analysis

Descriptive statistics were conducted to characterize the baseline UKB participants. Continuous variables were summarized as means and standard deviations, and categorical variables as frequencies and percentages. We constructed Cox proportional hazard regression models to estimate the HR and 95%CI among individuals with GSD compared to those without GSD for the time of follow-up. We used three sets of adjustments. Estimates in model 1 were adjusted only for sex, age, assessment center, and the top 40 genetic principal components. Estimates in model 2 were further adjusted for income, Townsend deprivation index, physical activity, smoking, drinking, sleep duration, hypertension, dyslipidemia, and waist-to-hip ratio (WHR). Estimates in model 3 were adjusted for, on top of model 2, type 2 diabetes mellitus (T2DM), and body mass index (BMI). In addition, a sensitivity analysis without the exclusion of less than two years of follow-up or a diagnosis of CVD within two years after developing GSD was performed. Finally, we repeated all these in following analyses including (i) limiting the exposure to cholecystectomy alone; (ii) investigating the sex-specific relationship between GSD and CVD; (iii) ruling out the reverse-direction association between CVD and the risk of subsequent GSD. All analyses were conducted using SAS version 9.4 (SAS Institute, Cary, NC). A two-sided *P*-value of less than 0.05 was considered statistically significant.

## Results

### Meta-analysis

A total of 10 cohort studies (eight publications) were included in the analysis [4, 6–8, 41–44], with three studies examining the association between GSD and stroke, and eight studies investigating the relationship between GSD and CAD. The sample sizes ranged from 2,208 to 487,373 participants. These studies were conducted across different regions, including Asia (*n* = 4), the United States (*n* = 4), and Europe (*n* = 2). The follow-up periods for these studies varied from 5 to 32 years. Of the 10 included studies, seven were prospective cohort studies, while the remaining three were retrospective cohort studies. The diagnosis of GSD was established using self-report questionnaires and medical records. The quality assessment scores for these studies ranged between 7 and 9, indicating high quality (Additional file 1: Table S5).

**Fig. 2:**
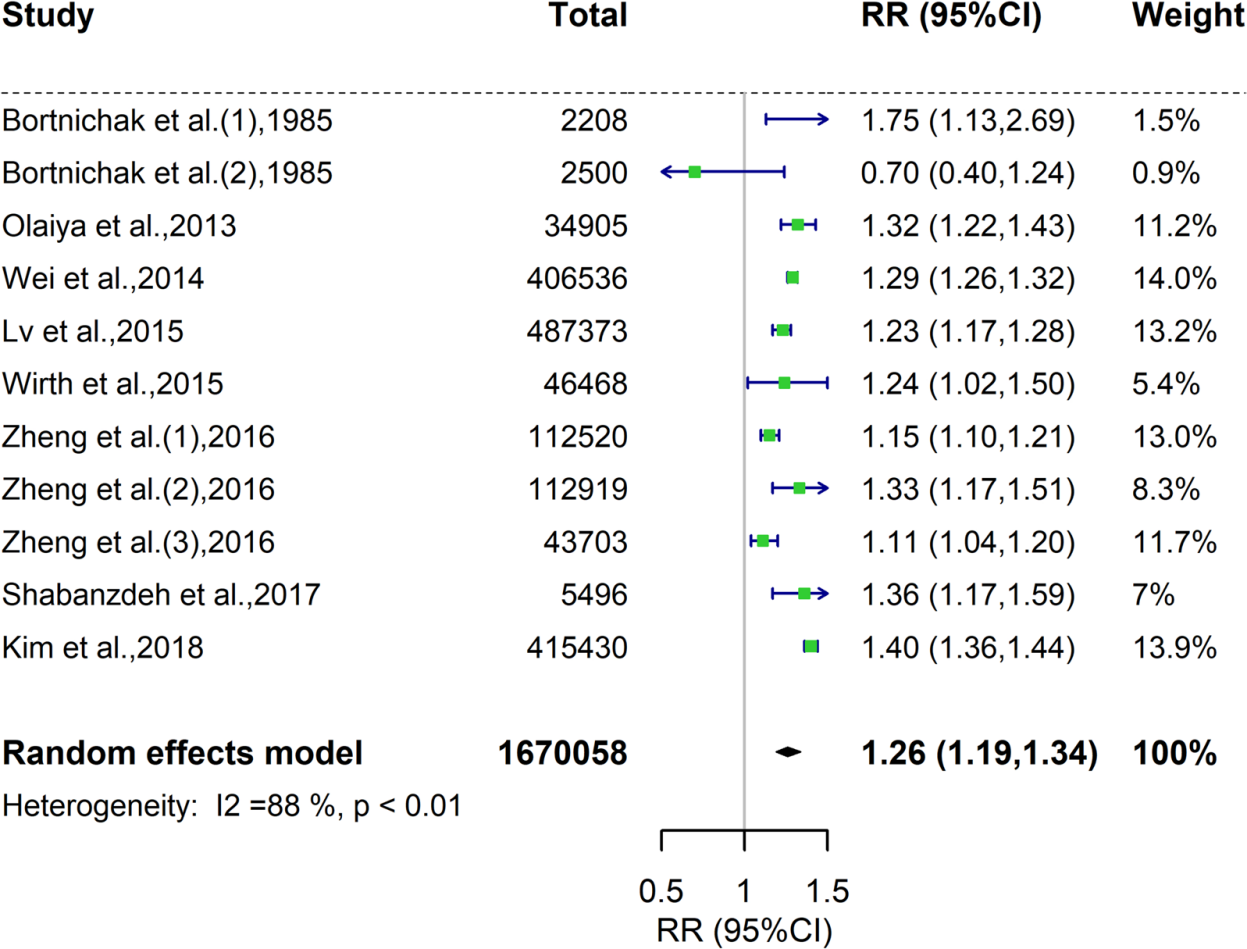
Forest plot of pooled relative risk of incident cardiovascular disease in participants with gallstone disease. Square represents the estimate of relative risk for each study; the horizontal line represents the 95% confidence intervals, and the diamond represents the overall estimate and its 95% confidence intervals. RR, relative risk. GSD, gallstone disease; cardiovascular disease (CVD)

Aggregating data from 8 existing cohort studies [4, 6–8, 41–44], the updated meta-analysis including over 1,670,058 participants derived a significant association (relative risk [RR] = 1.26, 95% confidence interval [CI] = 1.19–1.34), despite a pronounced heterogeneity (*P* < 0.01, I^2^ = 88%) (Fig. 2). Sensitivity analysis omitting one study at a time yielded similar findings, with RRs ranging from 1.24 to 1.28 (all *P* < 0.05) (data not shown). Specific to a particular CVD event, participants from 3 cohorts with a history of GSD had a 25% increased risk of stroke (RR = 1.25, 95% CIs = 1.17–1.35), whereas participants from 8 cohorts with a history of GSD had a 21% increased risk of CAD (RR = 1.21, 95% CIs = 1.13–1.30) (Additional file 2: Figure S1). In subgroup analyses, a marked reduction in heterogeneity was observed within the subgroups based on GSD diagnosis or study design (Additional file 1: Table S6). Thus, it is postulated that the heterogeneity may be attributed to GSD diagnosis and study design.

### Global and local genetic correlation

Both stroke (${r}_{g}$=0.16, *P* = 6.00 × 10^–4^) and CAD (${r}_{g}$=0.27, *P* = 2.27 × 10^–15^) showed a positive genetic correlation with GSD using LDSC, which remained consistent in GNOVA (GSD-stroke: ${r}_{g}$=0.14, *P* = 8.20 × 10^–6^; GSD-CAD: ${r}_{g}$=0.27, *P* = 7.89 × 10^–28^), all withstood Bonferroni correction (Table 1).

**Table 1:** Genome-wide genetic correlation between gallstone disease and cardiovascular diseases

**Models**	**Trait1**	**Trait2**	${{\varvec{r}}}_{{\varvec{g}}}$	${{\varvec{r}}}_{{\varvec{g}}}\_{\varvec{P}}$	***gcov***	***gcov_se***
LDSC	GSD	Stroke	0.16	6.00 × 10^–4^	0.011	0.005
		CAD	0.27	2.27 × 10^–15^	0.012	0.007
GNOVA	GSD	Stroke	0.14	8.20 × 10^–6^	0.009	0.002
		CAD	0.27	7.89 × 10^–28^	0.016	0.001

${r}_{g}$ genetic correlation, *GNOVA* genetic covariance analyzer, *LDSC* linkage disequilibrium score regression, *gcov* genetic covariance, *se* standard error, *GSD* gallstone disease, *CAD* coronary artery disease

**Fig. 3:**
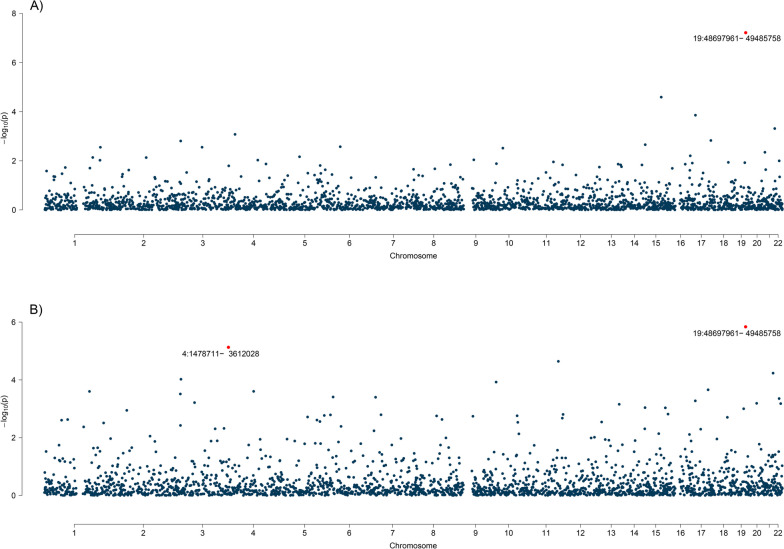
Local genetic correlation between gallstone disease and cardiovascular diseases. Manhattan plot presenting region-specific *P*-values for local genetic correlation between (**A**) gallstone disease and stroke and (**B**) gallstone disease and coronary artery disease. Red dots represent loci showing significant local genetic correlation after multiple testing adjustments (*P* < 0.05/2,353)

Partitioning the whole genome into LD-independent regions, only one significant region was observed for GSD and stroke (19q13.33, harboring *FUT2,* a previous-reported locus for GSD and stroke). With regard to CAD, two significant local regions were found, including 19q13.33 (the significant region reported in the GSD-stroke analysis) and 4p16.3 (harboring *HTT,* a previous-reported locus for myocardial infarction) [14]. All estimates withstood multiple corrections (*P* < 0.05/2,353) (Fig. 3 and Additional file 1: Table S7).

### Cross-trait meta-analysis and pleiotropic loci

**Fig. 4:**
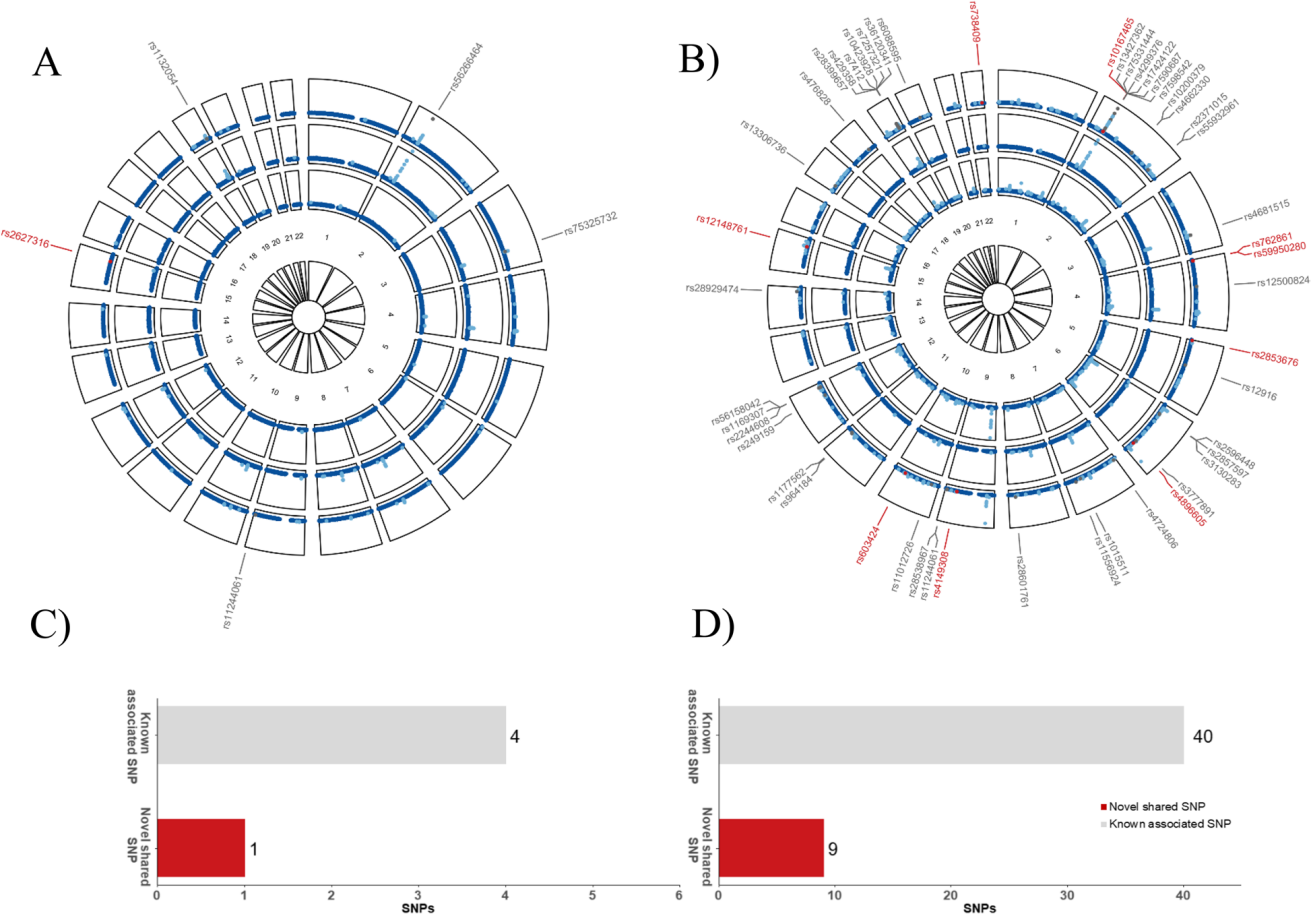
Cross-trait meta-analysis between gallstone disease and cardiovascular diseases. In each circular Manhattan plot, the outermost circle shows the cross-trait meta-analysis results between (**A**) gallstone disease and stroke and (**B**) gallstone disease and coronary artery disease; from the periphery to the center, each circle shows the GWAS results on gallstone disease and cardiovascular diseases, respectively. Light blue indicates variants with genome-wide significance (*P* < 5 × 10^–8^) while dark blue indicates variants with *P* ≥ 5 × 10^–8^. According to their single-trait and cross-trait characteristics, SNPs are divided into two different types named “Known associated SNP” and “Novel shared SNP”,which are presented in grey and red, respectively. RSIDs of them are listed. The bar plot presents the numbers of two types of SNPs detected in the cross-trait meta-analysis between (**C**) gallstone disease and stroke and (**D**) gallstone disease and coronary artery disease

In total, we identified five significant pleiotropic SNPs shared between GSD and stroke (Fig. 4 and Additional file 1: Table S8), among which one was novel. The significant novel shared SNP was rs2627316 (*P*_CPASSOC_ = 4.78 × 10^–8^) located near *ABHD17C,* a locus involved in protein depalmitoylation by enabling palmitoyl-(protein) hydrolase activity [45].

For GSD and CAD, we found a total of 49 significant pleiotropic SNPs among which nine were novel (Fig. 4 and Additional file 1: Table S9). The most significant novel shared SNP was rs59950280 (P_CPASSOC_ = 1.33 × 10^–10^) located near *HGFAC,* encodes a member of the peptidase S1 protein family, an enzyme activating hepatocyte growth factor (HGF), which further influence metabolic diseases and CVD [46]. The second most significant novel shared SNP was rs2853676 (P_CPASSOC_ = 4.52 × 10^–9^), mapped to *TERT*, a gene known to encode for telomerase reverse transcriptase, which was closely related to CAD [47, 48]. The last significant novel shared SNP rs4149308 (*P*_CPASSOC_ = 5.33 × 10^–9^) was located near *ABCA1,* a gene regulating cholesterol transporting [49], which was associated with GSD and CAD [50, 51].

Detailed annotations of each variant are shown in Additional file 1: Table S10-S11.

### Identification of causal variants and colocalization

For each CPASSOC-identified significant locus, we further determined a 99% credible set of causal SNPs using FM-summary (Additional file 1: Table S12-S13). In general, we found 37 candidate causal SNPs across all loci shared by GSD and stroke, and 771 candidate causal SNPs shared by GSD and CAD. Particularly, one SNP (rs11244061) was identified as having a posterior probability of 1.00 in the 99% credible set shared by GSD and stroke, and 11 SNPs (rs28929474, rs4299376, rs7598542, rs7412, rs13427362, rs17424122, rs7590687, rs429358, rs964184, rs59950280, and rs603424) shared by GSD and CAD. Notably, SNPs rs59950280 and rs603424 were also identified as novel shared loci in corresponding CPASSOC analysis.

We also performed colocalization analysis to determine whether pleiotropic SNPs driving the associations in two traits were the same or different. Fourteen of 49 shared loci colocalized at the same candidate causal SNPs for GSD-CAD while no colocalization was observed for GSD-stroke. (Additional file 1: Table S14-S15).

### Transcriptome-wide association study and shared genes

To investigate specific expression-trait associations that are likely shared by both traits, we further performed TWAS using gene expression data available at 49 tissues. Numbers of 48 shared gene-tissue pairs were identified for GSD and CAD, but none was identified for GSD and stroke (Additional file 1: Table S16). Most genes were identified specific to tissues of the digestive, endocrine, and cardiovascular systems. Among the number of 10 TWAS-significant genes, six were previously reported as susceptible to GSD and/or CAD (GWAS Catalog accessed by December 20, 2022), including *SPPL3*, *C12orf43*, *DAGLB*, and *UNC119B* associated with lipid metabolism (closely relevant to GSD and CAD), *SNRPD2* associated with CAD, and *RAC1* associated with CAD and lipid metabolism. Notably, all of these genes were located at pleiotropic loci identified in cross-trait meta-analysis, including *RAC1*, *FAM220A*, and *DAGLB* at 7p22.1; *SPPL3*, *C12orf43*, and *UNC119B* at 12q24.31; as well as SNRPD*2, DM1-AS* and *DMPK* at 19q13.32.

### Mendelian randomization analysis

**Fig. 5:**
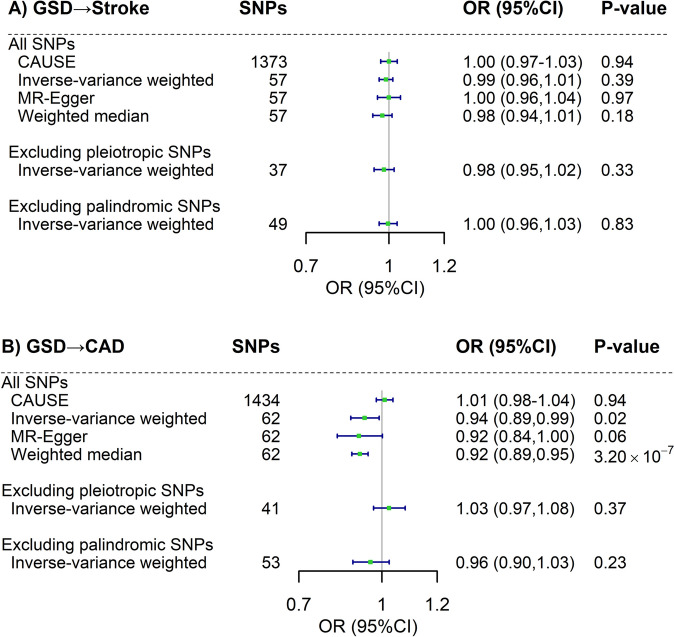
The estimated causal association between gallstone disease and cardiovascular diseases using two-sample Mendelian randomization. Boxes denote the point estimate of the causal effects between (**A**) gallstone disease and stroke and (**B**) gallstone disease and coronary artery disease. Error bars denote 95% confidence intervals. GSD, gallstone disease; CAD, coronary artery disease

We conducted a two-sample MR to make a causal inference. As shown in Fig. 5, no causal effect of genetically predisposed GSD on stroke was observed (OR_CAUSE_ = 1.00, 95%CI = 0.97–1.03). This estimate did not alter in IVW (OR = 0.99, 95%CI = 0.96–1.01), MR-Egger regression (OR = 1.00, 95%CI = 0.96–1.04) or weighted median approach (OR = 0.98, 95%CI = 0.94–1.01). Sensitivity analysis removing pleiotropic SNPs or palindromic SNPs revealed null findings. As for CAD, a consistent null result was observed (OR_CAUSE_ = 1.01, 95%CI = 0.98–1.04; OR_IVW_ = 0.94, 95%CI = 0.89–0.99; OR_MR-Egger_ = 0.92, 95%CI = 0.84–1.00; OR_weighted median_ = 0.92, 95%CI = 0.89–0.95; OR_pleiotropic IVs excluded_ = 1.03, 95%CI = 0.97–1.08; OR_palindromic Ivs excluded_ = 0.96, 95%CI = 0.90–1.03). In the reverse-direction MR, genetic predisposition to stroke or CAD did not seem to affect GSD risk (Additional file 2: Figure S2).

### Observational analysis

To explore whether phenotypic association consistent with the genetic relationship, we conducted an observational analysis leveraging the comprehensive UKB data. The baseline characteristics of UKB participants are presented in Additional file 1: Table S17-S18. In the analysis for the risk of incident stroke associated with GSD, participants were followed for 5,303,271 person-years (12.0 ± 2.0 years), during which 382 GSD patients and 7,224 GSD-free individuals developed stroke (Table 2). Consistent with MR results, we did not find any significant association between GSD and risk of stroke in the crude model (Mode 1 HR = 1.07, 95%CI = 0.97–1.19), neither in models where additional confounders were adjusted (Model 2 HR = 0.97, 95%CI = 0.85–1.11; Model 3 HR = 0.95, 95%CI = 0.83–1.09). Omitting the consideration of 2-year lag time (sensitivity analysis), however, we observed a significantly increased hazard of stroke among GSD patients (HR = 1.19, 95%CI = 1.06–1.34).

**Table 2:** Observational associations between gallstone disease and cardiovascular diseases

Exposure status	Cases/person-years	Model 1		Model 2		Model 3		Sensitivity analysis	
		**HR (95%CI)**	***P value***	**HR (95%CI)**	***P value***	**HR (95%CI)**	***P value***	**HR (95%CI)**	***P value***
**GSD → Stroke**									
No	7,224/5,095,745	1.00 (ref)		1.00 (ref)		1.00 (ref)		1.00 (ref)	
Yes	382/207,526	1.07 (0.97–1.19)	0.18	0.97 (0.85–1.11)	0.64	0.95 (0.83–1.09)	0.46	1.19 (1.06–1.34)	0.002
**GSD → CAD**									
No	24,709/4,810,395	1.00 (ref)		1.00 (ref)		1.00 (ref)		1.00 (ref)	
Yes	1,254/184,808	1.27 (1.20–1.35)	< 0.001	1.02 (0.95–1.10)	0.54	0.98 (0.91–1.06)	0.62	1.23 (1.15–1.31)	< 0.001

Model 1: adjusted for sex, age, assessment center, and the top 40 genetic principal components

Model 2: adjusted for sex, age, assessment center, the top 40 genetic principal components, income, Townsend deprivation index, physical activity (IPAQ), smoking, drinking, sleep duration, dyslipidemia, hypertension, and waist-to-hip ratio (WHR)

Model 3: adjusted for sex, age, assessment center, the top 40 genetic principal components, income, Townsend deprivation index, physical activity (IPAQ), smoking, drinking, sleep duration, dyslipidemia, hypertension, WHR, body mass index (BMI), Type 2 diabetes mellitus (T2DM)

*GSD* gallstone disease, *CAD* coronary artery disease, *HR* Hazard ratio, *CI* Confidence interval

In the analysis for the risk of incident CAD associated with GSD, participants were followed for 4,995,202 person-years (11.8 ± 2.3 years), during which 1,254 GSD patients and 24,709 GSD-free individuals developed CAD (Table 2). After adjusting for sex, age, assessment center, and the top 40 genetic principal components, GSD patients showed a significantly increased hazard of CAD (HR = 1.27, 95%CI = 1.20–1.35). With further adjustment in subsequent models, the effect diminished to null (Model 2 HR = 1.02, 95%CI = 0.95–1.10; Model 3 HR = 0.98, 95%CI = 0.91–1.06), despite sensitivity analysis showing a significant association in the fully adjusted model (HR = 1.23, 95%CI = 1.15–1.31).

Restricting exposure to cholecystectomy, we observed consistent null results (Additional file 1: Table S19). Similarly, sex-specific analysis yielded consistent findings (Additional file 1: Table S20). In the reverse-direction analysis, an independent effect of CAD but not stroke on GSD was observed (Additional file 1: Table S21).

## Discussion

Based on the significant association derived from our updated meta-analysis, we conducted comprehensive genetic and observational analyses to systematically investigate the shared genetic architecture and the phenotypic association between GSD and CVD (stroke and CAD). From a genetic perspective, our work demonstrated biological links underlying these complex traits, highlighting pleiotropy rather than causality. From a phenotypic perspective, the absence of associations between GSD and CVD risk further corroborates MR findings. Our work advances understanding to the complicated relationship underlying GSD and CVD, providing important implications for the prevention and treatment of the two common conditions.

GSD and CVD shared a moderate global genetic basis in our study. Extending to local level, we further identified two significant local signals (19q13.33 and 4p16.3) for GSD and CVD. Interestingly, the findings of SUPERGNOVA and CPASSOC analyses simultaneously discovered four pleiotropic genes (*SULT2B1 and FAM83E,* situated in the region 19q13.33; *RGS12 and HGFAC,* located in the region 4p16.3). These findings shed light on the shared biological mechanisms underlying GSD and CVD, encompassing cholesterol metabolism [52], protein signaling [53], and protease activation [46]. The intrinsic connection between GSD and CVD reflected by the significant global and local genetic correlations can be the result of shared biological mechanisms (pleiotropy) and/or causal associations (causality). In our downstream MR analysis (the first GSD-CVD MR to date) performed to explore these alternatives, we identified no causal relationship. Meanwhile, based on the large-scale rich data from the prospective UKB cohort, we found no phenotypic association between GSD and CVD either, which collided with previous observational studies supporting an overall significant association. For example, our meta-analysis aggregating data from 8 cohort studies reported an increased risk of CVD events among GSD patients (pooled RR = 1.26, 95% CI = 1.19–1.34). One potential explanation of such discrepancy is reverse causality, for which prior studies rarely considered. By integrating evidence from various sensitivity analyses that account for reverse causality (including omitting 2-year lag time and reverse-direction analysis), our findings largely imply reverse causation as a source of bias in previous significant results of observational studies and highlights the importance of temporal relationships in epidemiological designs. An alternative interpretation is that most of the previous studies failed to fully adjust for common confounders, especially for important metabolic factors such as BMI, dyslipidemia, T2DM, and hypertension that often co-exist with both GSD and CVD [6–8, 41]. After a careful enrollment and adjustment on a wider range of established and potential confounders, the GSD-CVD effect attenuated to null in our observational analysis, highlighting a non-trivial influence from residual confounding. Collectively, our work demonstrates a negligible causal association. Future investigations are warranted to further establish or rule out our findings.

Contrary to the limited evidence observed for causal relationships, we identified multiple pleiotropic loci, suggesting that the previously reported phenotypic links could be largely explained by common biological mechanisms. Here, we highlight two interesting examples. *ABHD17C*, as the only significant novel pleiotropic locus shared by GSD and stroke, involved in synapse development and synaptic plasticity by regulating protein depalmitoylation, further affecting neuronal system disease [45, 54]. Furthermore, *ABHD17C* is also associated with blood pressure, which may potentially elucidate the common mechanisms underlying GSD and CVD [55]. *HGFAC* was mapped by the most significant novel shared pleiotropic loci of GSD and CAD (index SNP: rs59950280), which was further corroborated by the findings of local genetic correlation (harbored in significant local region 4p16.3) and fine mapping analysis (having a posterior probability of 1.00 in the 99% credible set). It encodes a member of the peptidase S1 protein family. Initially, the protein is synthesized as an inactive single-chain precursor and subsequently undergoes endoproteolytic processing to be activated in a heterodimeric configuration. It acts as serine protease that converts hepatocyte growth factor to the active form. The increased circulation of HGF has been reported to be related to a wide variety of CVDs and metabolic disease [46, 56].

On a gene-tissue pair level, our TWAS analysis further revealed shared biological hypotheses between GSD and CAD. The three loci (7p22.1, 12q24.31, and 19q13.32) identified in both CPASSOC and TWAS analysis implicate common biological mechanisms in GSD and CVD, involving lipid metabolism [57, 58], inflammatory responses [59], and cell signaling [60]. In addition to the digestive and cardiovascular systems where the pathophysiology is well-documented, the identification of enrichment in endocrine systems (e.g., pituitary and thyroid) from TWAS suggests the possibility of shared pathways extending to a wider range of organs. The hormones including Thyroxine (T4), Triiodothyronine (T3), and Thyroid-stimulating Hormone (TSH) secreted by the thyroid/pituitary glands may partially elucidate the underlying mechanisms. On the one hand, the levels of free thyroxin and thyroid-stimulating hormone have been reported as markers of cardiovascular hemodynamic instability, which is closely related to CVDs such as CAD and heart failure [61]; on the other hand, hyperthyroidism is also assumed to be a risk factor for GSD in animal models and case report [62], more studies are needed to clarify the role of these hypothesized mechanisms., Therefore, rational intervention in the endocrine system may also facilitate the treatment of GSD and CVD in clinical practice.

Taken together, our findings deliver important clinical and public health implications. First, we provide evidence suggesting that GSD is very unlikely to pose a direct effect on the risk of CVD, implying that the treatment of GSD may not confer additional substantial cardiovascular health benefits, which partly supports the previous findings on that individuals with gallbladder removal did not benefit from a lowered CVD risk [6]. From a broader public health perspective, risk assessment of CVD in GSD patients is necessary, yet our study suggests it should be done in the same way as for the general population. Second, our genetic work demonstrates shared biological mechanisms underlying GSD and CVD. Prospectively, the identification of specific pleiotropic variants and pathways regulating common pathological elements may help discover therapeutic targets that would benefit both the prevention and treatment of GSD-CVD comorbidities. We hypothesize that aggregating large-scale GWAS to identify shared genetic underpinnings may guide the development of novel drugs or drug repurposing in the future.

Several limitations need to be acknowledged. First, the characteristics of GSD vary across races [63]. However, our findings were restricted to European ancestry population, limiting generalizability to other ethnicities. For instance, contrary to European populations, the benefit impact of gallbladder therapy on CVD has been observed in Asian populations [64]. Additionally, sex-specific genetic analysis was also hampered due to limited GWAS data availability. Nevertheless, based on our phenotypic analyses, it appears that sex heterogeneity may not exert a substantial influence. Incorporating sex-specific GWAS data would be beneficial for future investigations. Second, additional stroke subtypes were not included in our study due to the limited sample size; for instance, the cases of large artery stroke analyzed by Mishra A et al. (*N* < 10,000) [15]. Future studies are warranted with a larger sample size of the subtype-specific stroke. Third, the power of our MR analyses could still be limited by sample size, case proportion, and heritability of IVs, causing the overall null findings. However, by utilizing the most updated GWAS, our overall statistical power was considerably improved. We had 80% power at an $\alpha$-level of 0.05 to detect an association of 18% change for the risk of stroke and 2% change for the risk of CAD with GSD. Larger GWAS data are needed to validate our results in the future.

## Conclusions

In conclusion, leveraging large-scale population-based prospective observational studies and summary statistics from the hitherto largest GWAS, our study demonstrates an intrinsic link underlying GSD and CVD (stroke and CAD). While GSD is not likely to elevate the risk of stroke and CAD, it shares biological mechanisms with these conditions. Clinically, the treatment of GSD may not directly confer additional substantial cardiovascular health benefits. Further studies are needed to confirm our findings and to clarify potential mechanistic pathways.

### Supplementary Information

**Additional file 1:** **Table S1.** Characteristics of genetic instruments of gallstone disease. **Table S2. **Data sources, sample sizes, number of IVs. **Table S3.** The detailed search strategy for meta-analysis. **Table S4. **STROBE-MR checklist of recommended items to address in reports of Mendelian randomization studies. **Table S5. **Characteristics of included studies in meta-analysis. **Table S6.** Subgroup analyses for relationship between GSD and CVD. **Table S7.** Significant local regions after correcting for multiple testing (P < 0.05/2,353). **Table S8.** Genome-wide significant SNPs identified by cross-trait meta-analysis between gallstone disease and stroke (P-CPASSOC < 5×10^-8^, single trait *P*-value < 1×10^-3^, clumping r^2^=0.2). **Table S9.** Genome-wide significant SNPs identified by cross-trait meta-analysis between gallstone disease and coronary artery disease (P-CPASSOC < 5×10^-8^, single trait *P*-value < 1×10^-3^, clumping r^2^=0.2). **Table S10.** Linear closest genes of each cross-trait analyses-identified SNP for gallstone disease and stroke. **Table S11.** Linear closest genes of each cross-trait analyses-identified SNP for gallstone disease and coronary artery disease. **Table S12.** List of SNPs in the 99% credible set identified from fine-mapping analysis for each CPASSOC-identified locus shared between gallstone disease and stroke. **Table S13.** List of SNPs in the 99% credible set identified from fine-mapping analysis for each CPASSOC-identified locus shared between gallstone disease and coronary artery disease. **Table S14.** Results from colocalization analysis for each pleiotropic locus identified from CPASSOC between gallstone disease and stroke. **Table S15.** Results from colocalization analysis for each pleiotropic locus identified from CPASSOC between gallstone disease and coronary artery disease. **Table S16.** Significant shared transcriptome-wide association analysis results between gallstone disease and coronary artery disease. **Table S17.** Baseline characteristics of UK Biobank participants by gallstone disease status during follow-up for stroke. **Table S18.** Baseline characteristics of UK Biobank participants by gallstone disease status during follow-up for coronary artery disease. **Table S19.** Observational associations between cholecystectomy and cardiovascular diseases. **Table S20.** Sex-specific observational associations between gallstone disease and cardiovascular diseases. **Table S21.** Observational associations between cardiovascular diseases and gallstone disease.

**Additional file 2:** **Figure S1.** Forest plot of pooled relative risk of incident stroke and CAD in participants with GSD. **Figure S2.** Estimated causal association between cardiovascular diseases and gallstone disease using two-sample Mendelian randomization.

## Data Availability

The UK Biobank analysis was conducted with the application 50538. GWAS summary statistics for stroke are publicly available from the NHGRI-EBI GWAS catalog (https://www.ebi.ac.uk/gwas/, accession codes: GCST90104539 (https://www.ebi.ac.uk/gwas/studies/GCST90104539). GWAS summary statistics for CAD are publicly available from the NHGRI-EBI GWAS catalog (https://www.ebi.ac.uk/gwas/, accession codes: GCST90132314 (https://www.ebi.ac.uk/gwas/studies/GCST90132314).

## Abbreviations

GSD: Gallstone disease
CVD: Cardiovascular disease
CAD: Coronary artery disease
UKB: UK Biobank
RR: Relative risk
CI: Confidence interval
OR: Odds ratio
HR: Hazard ratio
SNP: Single-nucleotide polymorphism
GWAS: Genome-wide association study
IV: Instrumental variable
MR: Mendelian randomization
ICD: International Classification of Diseases
OPCS: Operative Procedures
$${r}_{g}$$: Genetic correlation
LDSC: Linkage disequilibrium score regression
GNOVA: Genetic covariance analyzer
CPASSOC: Cross-Phenotype Association
VEP: Ensembl Variant Effect Predictor
PPA: Posterior probabilities of association
TWAS: Transcriptome-wide association study
GTEx: Genotype-Tissue Expression
CAUSE: Causal Analysis Using Summary Effect Estimates
IVW: Inverse-variance weighted
WHR: Waist-to-hip ratio
T2DM: Type 2 diabetes mellitus
BMI: Body mass index
HGF: Hepatocyte growth factor
T4: Thyroxine
T3: Triiodothyronine
TSH: Thyroid-stimulating Hormone
