# Supplementary material for: Impact of gallstone disease on the risk of stroke and coronary artery disease: evidence from prospective observational studies and genetic analyses
Source: BMC Med. 2023 Sep 13;21:353. doi: 10.1186/s12916-023-03072-6 (PMC10500913; doi:10.1186/s12916-023-03072-6)
Supplement: Supplementary file 2 — Additional file 2: Figure S1. Forest plot of pooled relative risk of incident stroke and CAD in participants with GSD. Figure S2. Estimated causal association between cardiovascular diseases and gallstone disease using two-sample Mendelian randomization. [file 12916_2023_3072_MOESM2_ESM.docx]

**Additional file 2**


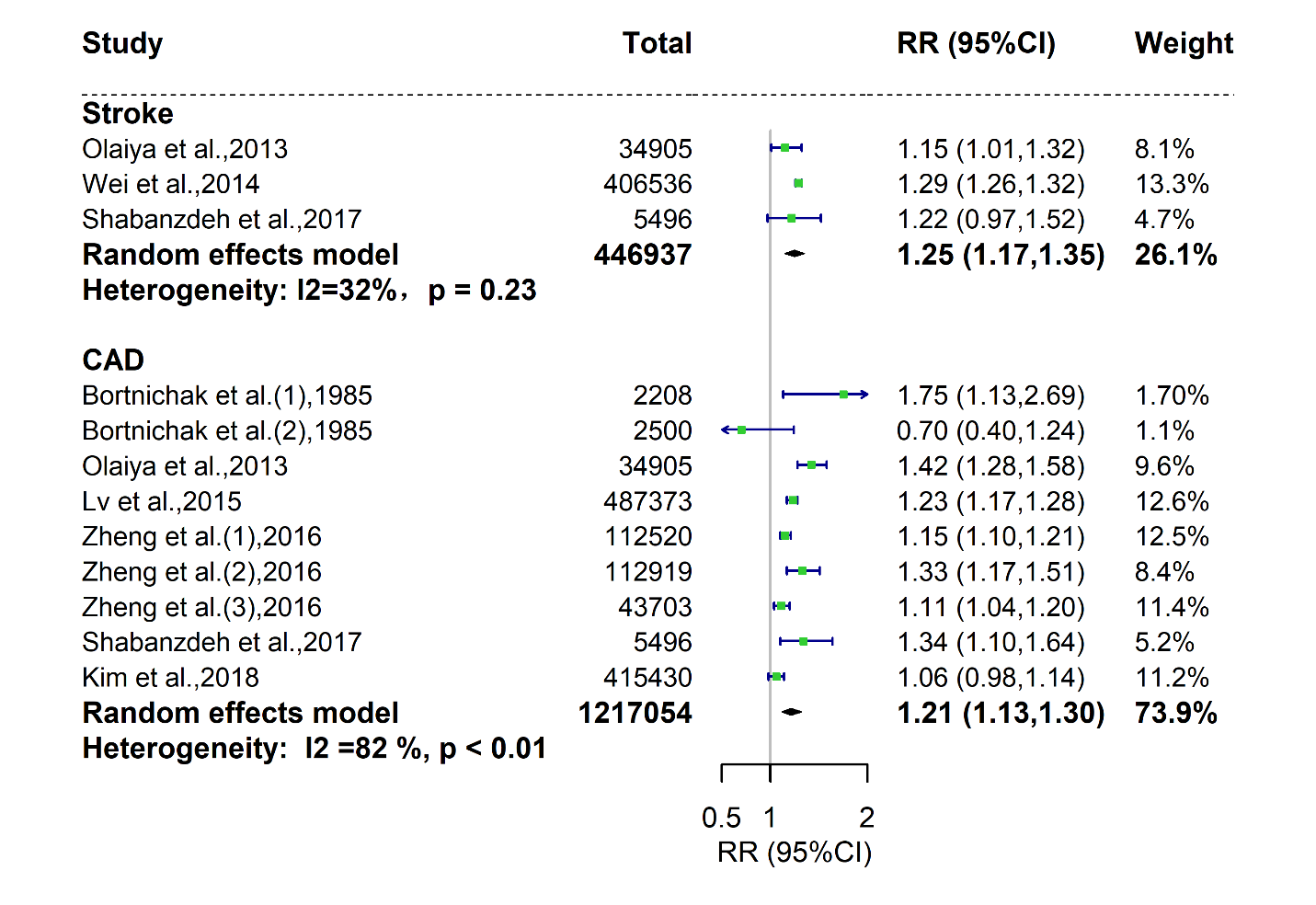


**Figure S1.** Forest plot of pooled relative risk of incident stroke and CAD in participants with GSD. Square represents the estimate of rate ratio for each study; horizontal line represents the 95% confidence intervals, and diamond represents the overall estimate and its 95% confidence intervals. RR, rate ratio. GSD, gallstone disease; CAD, coronary artery disease.


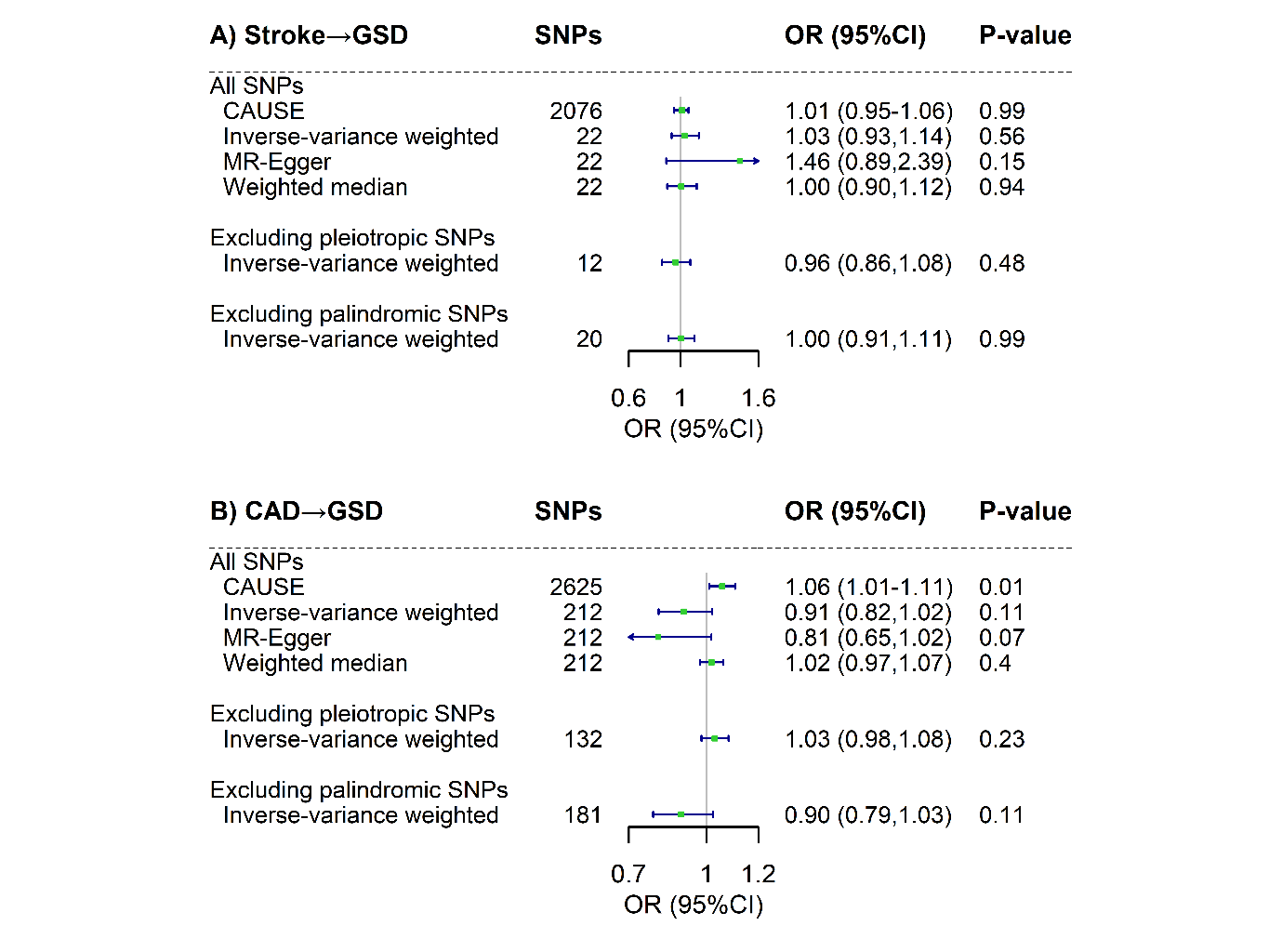


**Figure S2.** Estimated causal association between cardiovascular diseases and gallstone disease using two-sample Mendelian randomization. Boxes denote the point estimate of the causal effects between (A) stroke and gallstone disease, (B) coronary artery disease and gallstone disease. Error bars denote 95% confidence intervals. GSD, gallstone disease; CAD, coronary artery disease.
